# 316L Stainless Steel Thin-Walled Parts Hybrid-Layered Manufacturing Process Study

**DOI:** 10.3390/ma16196518

**Published:** 2023-09-30

**Authors:** Xuefeng Wu, Chentao Su, Kaiyue Zhang

**Affiliations:** School of Mechanical and Power Engineering, Harbin University of Science and Technology, Harbin 150080, China; su2783108489@163.com (C.S.); 15512374573@163.com (K.Z.)

**Keywords:** additive and subtractive hybrid manufacturing, thin-walled parts, process study, 316L stainless steel

## Abstract

Additive manufacturing technology overcomes the limitations imposed by traditional manufacturing techniques, such as fixtures, tools, and molds, thereby enabling a high degree of design freedom for parts and attracting significant attention. Combined with subtractive manufacturing technology, additive and subtractive hybrid manufacturing (ASHM) has the potential to enhance surface quality and machining accuracy. This paper proposes a method for simulating the additive and subtractive manufacturing process, enabling accurate deformation prediction during processing. The relationship between stress distribution and thermal stress deformation of thin-walled 316L stainless steel parts prepared by Laser Metal Deposition (LMD) was investigated using linear scanning with a laser displacement sensor and finite element simulation. The changes in stress and deformation of these thin-walled parts after milling were also examined. Firstly, 316L stainless steel box-shaped thin-walled parts were fabricated using additive manufacturing, and the profile information was measured using a Micro Laser Displacement Sensor. Then, finite element software was employed to simulate the stress and deformation of the box-shaped thin-walled part during the additive manufacturing process. The experiments mentioned were conducted to validate the finite element model. Finally, based on the simulation of the box-shaped part, a simulation prediction was made for the box-shaped thin-walled parts produced by two-stage additive and subtractive manufacturing. The results show that the deformation tendency of outward twisting and expanding occurs in the additive process to the box-shaped thin-walled part, and the deformation increases gradually with the increase of the height. Meanwhile, the milling process is significant for improving the surface quality and dimensional accuracy of the additive parts. The research process and results of the thesis have laid the foundation for further research on the influence of subtractive process parameters on the surface quality of 316L stainless steel additive parts and subsequent additive and subtractive hybrid manufacturing of complex parts.

## 1. Introduction

316L stainless steel is an austenitic stainless-steel material containing a certain number of microelements, such as Cr and Ni, and has good thermal plasticity and wear resistance. It is widely used to manufacture precision equipment, such as aviation engine parts, and transportation and electronic communication parts [1,2]. Thin-walled parts refer to workpieces whose wall thickness to profile size ratio does not exceed 1:20. Manufacturing thin-walled parts has always been a key research topic in traditional manufacturing industries. The clamping structure and clamping force, tool selection, cutting process parameters, and other factors have a significant impact on thin-walled parts. Moreover, the production of thin-walled components is also limited by factors such as inadequate cutting rigidity and vibration during machining. Additive manufacturing (AM), also known as 3D printing, is defined as the “process of joining materials to make objects from 3D model data usually layer upon layer, as opposed to traditional manufacturing technologies such as subtractive manufacturing” [3]. This unique manufacturing method has created enormous potential for producing parts with complex geometries while reducing material waste and shortening the time to market [4]. Different types of AM technologies have been developed, which can be classified according to different factors, including raw materials and building methods [5].

Due to its advantages in manufacturing complex geometries and reducing material waste, AM has been applied in many fields [6]. However, some AM limitations have hindered its comprehensive application in different industries. For example, AM parts typically have lower accuracy and strength [7], poor surface quality [8], and longer manufacturing times [9]. Therefore, improving AM processes’ size and mechanical properties is necessary while shortening production time [10]. At the same time, AM still faces some technical bottlenecks, such as internal stresses caused by transient melting/solidification of the cladding layer during laser additive manufacturing, which seriously affects the geometric dimensions and mechanical properties of the parts, leading to severe deformation and cracking of the parts [11]. After a single-layer laser scan, there are high residual tensile stresses in the solidified layer, and the internal stresses accumulate continuously after multiple layers are stacked, making the parts prone to deformation and cracking [12,13], thereby seriously affecting the strength and forming accuracy of the parts. These are the problems that urgently need to be solved in additive manufacturing.

Additive/Subtractive Hybrid Manufacturing (ASHM) is an emerging manufacturing technology that combines the advantages of Additive Manufacturing (AM) and traditional Subtractive Manufacturing (SM) [14,15]. This technology relies on Laser Metal Deposition (LMD), which combines AM and SM and can produce parts with complex geometries, as well as good surface finish and dimensional accuracy [16]. In ASHM-based Selective Laser Melting (SLM), after the scanning laser beam forms multiple powder layers, the milling cutter machines the finished parts, and the next SLM process begins. The additive and subtractive processes are repeated alternately until the part is manufactured [17]. ASHM is one of the most effective methods to improve the surface quality and dimensional accuracy of complex AM samples [18,19,20]. The mixed additive and subtractive manufacturing process is gaining popularity as an effective solution to overcome the limitations of AM while also enhancing the dimensional accuracy and surface quality of parts [21].

Hybrid-layered manufacturing refers to the alternating use of additive manufacturing and subtractive machining without changing the workpiece’s position, fixture, or reference surface, thereby reducing machining errors caused by repeated positioning. Its advantages are particularly evident in processing thin-walled parts with complex curved surfaces, internal structures, and enclosed cavities. Processing thin-walled parts with complex curved surfaces and internal structures poses significant challenges for traditional machining methods. However, hybrid-layered manufacturing technology optimizes the benefits of additive and subtractive manufacturing by initially milling the inner cavity of the part, followed by a second additive manufacturing step. This approach circumvents tool–workpiece interference and addresses the issue of inaccessible cavity regions during machining; therefore, hybrid-layered manufacturing technology is considered an efficient and precise machining method that can greatly improve production efficiency and reduce costs.

Yang et al. [22] conducted additive/subtractive hybrid manufacturing experiments on 316L stainless steel using an integrated additive DED process and CNC milling machine. Different laser powers (*p*), scanning speeds (*v*), and laser scanning paths were used in the experiments. The results showed that the depth and width of the additive melt pool were significantly affected by p, v and the additive parts’ mechanical properties exhibited anisotropy in different printing directions. Gong et al. [23] also used the DED process combined with CNC milling to conduct ASHM experiments on 316L stainless steel. The finished parts were machined after deposition, and various deposition paths were used to study the influence of deposition paths on the final part quality. The hardness and mechanical properties test results showed that the “S”-type deposition method obtained higher hardness, yield strength (479 MPa) under tensile loading, and ultimate tensile strength (688 MPa). Zhao et al. [24] systematically studied the microstructure and properties of WC particle-reinforced 316L stainless steel produced by additive/subtractive hybrid manufacturing. The effects of process parameters on part properties were comprehensively described from aspects such as density, microstructure, hardness, and surface quality. The results showed that laser power and scanning speed had a particularly significant impact on part properties. Wei Du et al. [25] conducted additive/subtractive hybrid manufacturing on 18Ni-300 maraging steel samples. The results showed that the selective laser melting (SLM) sample had a finer cellular microstructure with dendritic extensions than the forged sample. The ASHM samples had a 31.6% higher hardness than the forged samples and exhibited directionality independence. Under the same cutting conditions, the cutting force of the SLM samples was higher than that of the forged samples. At a high feed rate of 320 mm/min, the difference in cutting force between the two samples increased to 29.8%. Chen F et al. [26] conducted powder-fed laser additive manufacturing and milling subtractive manufacturing. The additive and milling processes alternated during manufacturing, with milling performed every 2 mm of deposition until the entire sample was completed. The subsequent analysis of the workpiece detection results showed that the surface microhardness of the ASHM samples was 11.2% and 33.7% higher than that of the AM samples and forged parts, respectively. The tensile strength and yield strength under tensile loading were increased by about 5% and 60.5%, respectively, while the elongation at break was slightly reduced.

In 2018, Lockheed Martin used electron beam additive and subtractive manufacturing technology to produce the largest 3D-printed space component to date—a satellite fuel tank. Utilizing this process, the component achieved over 80% reduction in material waste and reduced the overall delivery time from two years to three months. The part underwent rigorous quality testing, enabling it to withstand the harsh demands of launch and perform reliably in the vacuum of space for several decades. Currently, this component is being utilized as a standard part of the LM 2100 satellite platform.

DMG MORI’s unique hybrid (combining additive manufacturing with traditional manufacturing) machining solution is a remarkable technology that integrates milling techniques with laser metal deposition processes. It is applied on a laser deposition machine tool that possesses complete milling capabilities. In May 2019, Virgin Orbit, a satellite launch company, utilized DMG MORI’s additive and subtractive integrated equipment to 3D print engine combustion chambers for NASA. The interior lining of the chambers was made of GRCop-84 copper alloy, while the exterior was composed of nickel-based high-temperature alloy. The structure and materials were identical to traditional designs, but the manufacturing time was reduced by several months.

The study of the additive and subtractive hybrid manufacturing process with alternating increments and reductions is difficult and complex. Currently, most research focuses on the post-additive/subtractive manufacturing process, where the material is first added and then subtracted. In alternating between additive and subtractive manufacturing, it is essential to consider not only the impact of these processes on part quality but also factors such as thermal cutting, the timing of additive and subtractive steps, and temperature distribution across additively manufactured parts during cutting. Currently, there is a relative lack of research in this area, thus necessitating an investigation into the process of alternating additive and subtractive manufacturing.

In this study, we investigate the utilization of additive and subtractive manufacturing tools to evaluate the additive and subtractive processes for thin-walled box components along two distinct paths. The experimental process and resulting data were acquired through a laser displacement sensor, which effectively characterized the deformation patterns exhibited by the box-shaped thin-walled part during additive/subtractive processing with different paths, thereby elucidating the underlying causes for deformation patterns.

**Figure 1:**
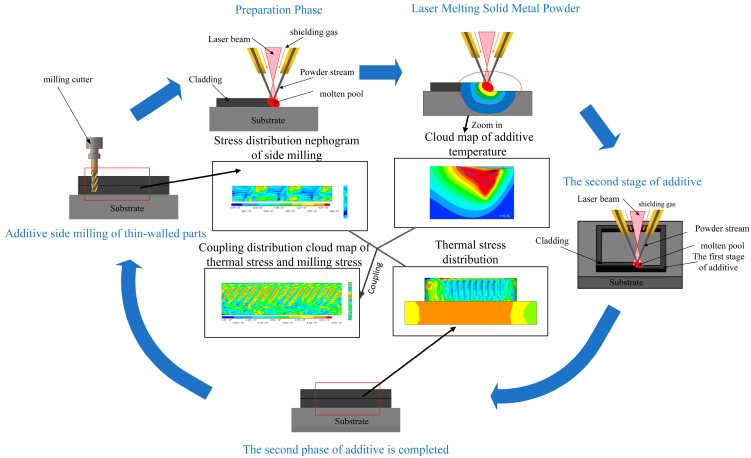
Schematic diagram of the forming process of the hybrid processing parts.

In this article, the deposition manufacturing of a thin-walled part is carried out on a rectangular substrate. First, the laser beam generates a melt pool on the substrate surface, and then the metal powder is sprayed into the melt pool through a powder feeder, forming the first layer. As the laser beam scans layer by layer, the melt pool gradually moves upward. The powder is sprayed into the melt pool and gradually melts, forming a new melt pool. In this new melt pool, the laser beam continues to scan and melt the powder, thus adding material layer by layer. After depositing 20 layers, the surface of the additive manufacturing part is milled to improve the surface quality. The above operations are repeated until the box-shaped thin-walled part is completed. The schematic diagram of the part-forming process is shown in Figure 1.

## 2. Theoretical Analysis of Additive and Subtractive Hybrid Manufacturing Process

### 2.1. Heat Transfer Analysis of Additive Hybrid Manufacturing Process

During the laser additive manufacturing process, the melted metal powder is layered and deposited on the substrate as the high-energy laser beam moves back and forth. This process involves melting and solidifying the metal material, transforming the metallographic structure, etc. For isotropic materials, the heat transfer behaviors, such as conduction, convection, and thermal radiation among the melt pool, cladding layer, substrate, and the surrounding air, can be described by the heat transfer differential equations.

(1) $$\rho c\frac{\partial T}{\partial t}=\frac{\partial }{\partial x}\left(\lambda_{x}\frac{\partial T}{\partial x}\right)+\frac{\partial }{\partial y}\left(\lambda_{y}\frac{\partial T}{\partial y}\right)+\frac{\partial }{\partial z}\left(\lambda_{z}\frac{\partial T}{\partial z}\right)+Q$$

where ρ is the material density, $\mathrm{kg}/m^{3}$; c is the material specific heat capacity, $J/\left(\mathrm{kg}\cdot K\right)$; T denotes the material transient temperature, K; t denotes time, s; and $\lambda_{x}$, $\lambda_{y}$, and $\lambda_{z}$ denote the thermal conductivity in the x, y, and z directions, $W/\left(m\cdot K\right)$. When the material is isotropic, $\lambda_{x}=\lambda_{y}=\lambda_{z}=\lambda$.

The thermal boundary conditions in the additive process include the laser heat source, the convection of the cladding layer in contact with the air, and the thermal radiation boundary. A two-dimensional Gaussian heat source is used to describe the laser energy distribution heat source, and the maximum heat flow density at the center of the laser spot is:

(2) $$q_{m}=(3\cdot k\cdot U\cdot I)/(3.14\cdot r^{2})$$

where k is the thermal efficiency; U∗I is the laser power; and r is the spot diameter. Combined with the Gaussian function so that the volumetric heat generation rate is Gaussian distributed within the cell, the Gaussian heat source used in the numerical simulation is defined by the following equation:

(3) $$HGENr=\left[q_{m}\cdot \exp(-3.14\cdot r^{2}/4)\right]/L$$

where L is the thickness per unit volume and r is the incremental spot diameter; the heat flow rate decreases with increasing diameter, according to the grid model to choose the initial value, wherein the initial value in this paper is 1 and the increment is 0.5.

The convective heat transfer between a flowing fluid and a solid is proportional to its temperature difference and can be expressed by the following equation:

(4) $$q_{s}=h\cdot (T-T_{0})$$

where $q_{s}$ is the heat exchanged between the solid surface and fluid per unit area per unit time, called heat flow density, unit $W/m^{2}$; T is the solid surface temperature; $T_{0}$ is the fluid temperature; and $T_{m}$ is the convective heat transfer coefficient, with a natural convection for 5~25 and gas-forced convection for 25~300. The net radiation heat transfer rate equation can express thermal radiation:

(5) $$q_{r}=\varepsilon \delta (T^{4}-T_{0}^{4})$$

where $q_{r}$ is the heat emitted per unit area of the solid surface, with units of $W/m^{2}$; ε is the emissivity, typically ranging from 0 to 1; δ is the Stefan–Boltzmann constant, which is $5.67\times 10^{-8}\left(W/m^{2}\cdot K^{4}\right)$; T is the solid temperature; and $T_{0}$ is the ambient temperature. $T_{0}$ ensures the convergence of temperature calculation in the LMD additive forming process, the convective and radiative heat transfer are combined $\left(q=q_{s}+q_{r}\right)$, and a factor $\left(T-T_{0}\right)$ is introduced. This leads to the radiative–convective boundary heat transfer equation:

(6) $$q=\left[\varepsilon \delta (T^{2}+T_{0}^{2})\cdot (T+T_{0})+h\right]\cdot (T-T_{0})$$

### 2.2. Thermal Stress Analysis of Additive Hybrid Manufacturing Process

The LMD stress and deformation simulation is based on the numerical simulation results of the temperature field. By applying boundary conditions in static analysis, the software can read the temperature history of all nodes and calculate the resulting stress and deformation of the model.

Currently, the stress distribution in laser additive manufacturing is usually analyzed based on the theory of thermo-elasto-plasticity. In the stress–strain relationship of thermo-elasto-plastic mechanics, a term of strain caused by temperature change is added while considering the effect of temperature on yield stress, etc. Given that the stress changes in the LMD process are very complex, it is necessary to perform reasonable and moderate simplification when conducting thermo-elasto-plastic analysis. It is usually based on the assumptions that (1) the yield criterion for metallic materials is based on the Von–Mises criterion, and (2) the flow criterion and the hardening criterion are satisfied when the metallic material is in the plastic region.

According to the Von–Mises criterion, the component undergoes yielding when the equivalent stress exceeds the yield stress.

(7) $$\bar{\sigma }=\sqrt{\left[\left(\sigma_{x}-\sigma_{y})+(\sigma_{y}-\sigma_{z})+(\sigma_{z}-\sigma_{x})+6(\tau_{xy}^{2}+\tau_{yz}^{2}+\tau_{xz}^{2}\right)\right]/2}$$

where, $\sigma_{x}$, $\sigma_{y}$, $\sigma_{z}$ is the positive pressure in x, y, z direction; and $\tau_{xy}$, $\tau_{yz}$, $\tau_{xz}$ is the shear stress.

The flow criterion defines the magnitude and direction of the plastic strain components when the material undergoes plastic deformation. Its differential equation is as follows:

(8) $$dg=\frac{\partial g}{\partial \sigma_{ij}}d\sigma_{ij}+\frac{\partial g}{\partial \varepsilon^{p}{}_{ij}}d\varepsilon^{p}{}_{ij}+\frac{\partial g}{\partial k}dk$$

where $\sigma_{ij}$ is the total stress, $\varepsilon^{p}{}_{ij}$ is the total plastic strain, and k is the hardening parameter.

**Figure 2:**
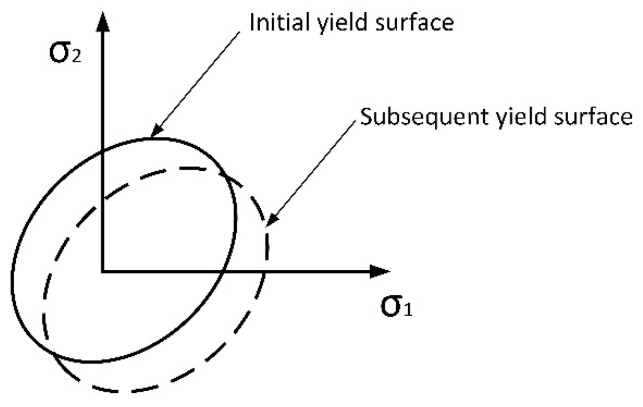
Kinematic hardening model.

The hardening criterion is used to describe the variations in the yield surface. In this model, known as the kinematic hardening model, the center of the yield surface moves along the yielding direction in stress space, while the size and shape of the yield surface remain unchanged, as shown in Figure 2.

The thermo-elasto-plastic equation is as follows:

(1) Stress–strain relationship

The equation for the stress–strain relationship when the material is in a thermo-elasto-plastic state is as follows:

(9) {dσ}=[D]{dε}−{C}dT

where $d_{\sigma }$ is the stress increment; $d_{\varepsilon }$ is the strain increment; dT is the temperature increment; $\left[D\right]$ is the elastic or elastoplastic matrix; and $\left\{C\right\}$ is the vector related to temperature.

In the elastic region:

(10) $$\{D\}={\left\{D\right\}}_{e},\{C\}={\left\{C\right\}}_{e}={\left[D\right]}_{e}\left(\left\{\alpha \}+\frac{\partial {\left[D\right]}_{e}^{-1}}{\partial T}\{\sigma \right\}\right)$$

where α is the coefficient of linear expansion and T is the temperature.

In the plastic region, it is assumed that the yield condition of the material is:

(11) $$f(\sigma )=f_{0}\left(\varepsilon_{p},T\right)$$

where f is the yield function and $f_{0}$ is a function of the yield stress related to temperature and plastic strain.

According to the plastic flow rule, the plastic strain increment $\left\{d\varepsilon_{p}\right\}$ has the following relationship:

(12) $$\left\{d\varepsilon_{p}\right\}=\lambda \left\{\frac{\partial f}{\partial \sigma }\right\}$$

The loading or unloading of the plastic region is judged by λ. Loading is indicated when λ>0, and unloading is indicated when λ<0.

(2) Equilibrium equation

For a numerical model unit of laser cladding, the following equilibrium equation holds:

(13) $${\left\{dF\right\}}^{e}+{\left\{dR\right\}}^{e}={\left[K\right]}^{e}{\left\{d\sigma \right\}}^{e}$$

(14) $${\left[K\right]}^{e}=\int {\left[B\right]}^{T}[D][B]dV$$

(15) $${\left\{dR\right\}}^{e}=\int \{{\left[B\right]}^{T}\{C\}dTdV$$

where ${\left\{dF\right\}}^{e}$ is the increment of force on the unit node; ${\left\{dR\right\}}^{e}$ is the increment of unit initial strain equivalent node force caused by temperature; ${\left\{d\sigma \right\}}^{e}$ is the increment of node displacement; ${\left[K\right]}^{e}$ is the stiffness matrix of the unit; and $\left[B\right]$ is the matrix linking the strain and nodal displacement vectors in the unit.

Based on the unit being in an elastic or plastic state, ${\left[D\right]}_{e}$ and ${\left\{C\right\}}_{e}$ replace $\left[D\right]$ and $\left\{C\right\}$ in the above equation to form the unit stiffness matrix and equivalent node load. Then, the total stiffness matrix $\left[K\right]$ and total load vector $\left\{dF\right\}$ are integrated to obtain the equilibrium equation set for the entire component:

(16) [K]{dσ}={dF}

where $\left[K]=\sum {\left[K\right]}^{e};\{dF\}=\sum ({\left\{dF\right\}}^{e}+{\left\{dR\right\}}^{e}\right)$.

Considering that there is generally no external force in the laser additive remanufacturing process, the corresponding nodal forces of the unit around each node are self-equalizing force systems, i.e., $\sum {\left\{dF\right\}}^{e}=0,\{dF\}=\sum {\left\{dR\right\}}^{e}$.

### 2.3. Thermal Stress Analysis of Subtractive Hybrid Manufacturing Process

**Figure 3:**
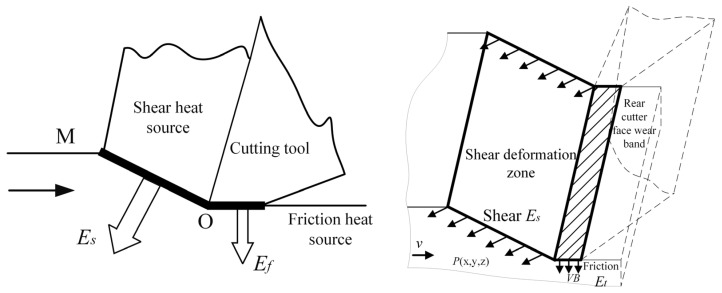
The influence of the heat source in the cutting zone on the temperature field of the workpiece surface.

In CNC milling, most energy expended during chip deformation is transformed into thermal energy. With an increased cutting speed, there is a corresponding increase in heat generation, thereby significantly affecting machining deformations [27]. The temperature distribution on the workpiece surface is primarily determined by the temperature fields at the shear plane and tool–workpiece interface in the third deformation zone. It is necessary to analyze separately the shear deformation energy $E_{s}$ generated by the heat source in the shear zone and the frictional work $E_{f}$ generated by the heat source in the friction zone formed by the tool flank tool face and the workpiece surface, as shown in Figure 3. It should be noted that although the contact surface temperature of the workpiece and the tool’s flank face obtained during the calculation process is the sum of several deformation zone temperatures, the contact zone is extremely small in length (assuming a maximum dullness value of $VB_{\max}0.3\;mm$ for the flank) and the cutting speed is very high. The tool moves quickly through the surface of the workpiece, and the surface of the workpiece has good heat dissipation. Therefore, the temperature of the machined surface of the workpiece is not very high and can be ignored.

### 2.4. Deformation Processing Method

The deformation model of a workpiece includes two parts: deformation caused by thermal stress and deformation caused by the interaction between the cutting tool and the workpiece. During the additive manufacturing process, due to the high temperature gradient and rapid heating–cooling process, thermal expansion and contraction cause materials to undergo uneven plastic deformation under constraints, generating high residual stress. During the subtractive manufacturing process, on the one hand, the residual stress generated during the additive manufacturing stage will redistribute as materials are removed, resulting in the deformation of the material; on the other hand, due to the thin wall thickness and poor stiffness of the part, deformation occurs under the cutting force of the cutting tool and the workpiece, causing the material removal amount to not reach the theoretical value and resulting in machining errors due to material rebound after processing. These two effects interact with each other, ultimately leading to part deformation.

The “birth–death element” technique is a commonly used numerical simulation method in additive manufacturing, which simulates the molted and to-be-molted parts of the additive process through the “birth” and “death” of the elements. In ANSYS, “dead” elements are not simply removed elements but rather have their stiffness matrix multiplied by a very small factor, which the program defaults to 1 × 10^−6^ (can be defined as needed). The mass, specific heat, and strain of the “dead” elements are all set to zero, and their mass and energy will not be included in the solution results.

**Figure 4:**
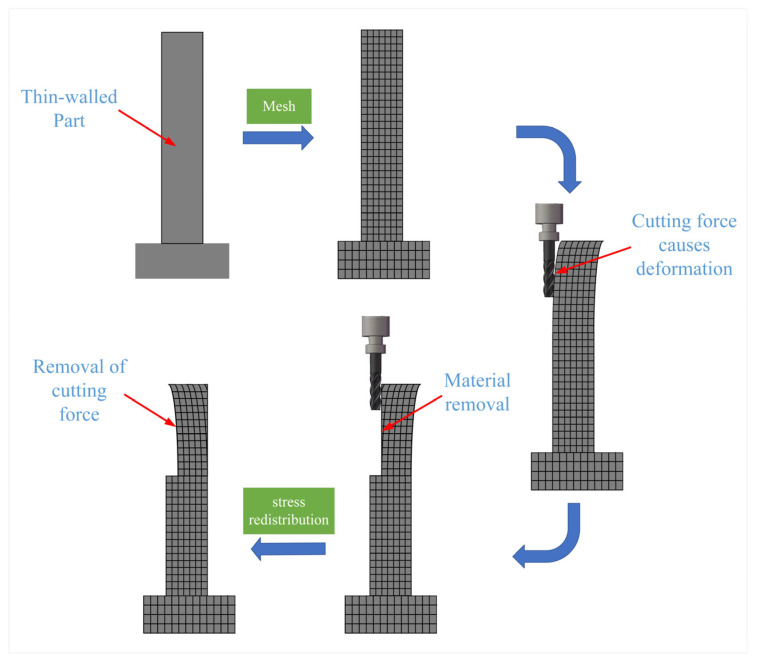
Milling diagram of thin-walled parts.

In the simulation process, the “birth–death element” technique is first used to simulate the additive process, and the material’s morphology after processing is obtained through thermal stress simulation. In the subtractive stage, three-dimensional cutting forces are applied to the mesh positions, causing deformation of the thin-walled part. Material is removed by selecting the coordinate values in the y direction and killing the corresponding elements. After killing, the solution is obtained, and the stress is redistributed, resulting in the final deformation result of the workpiece. The schematic diagram of the milling of thin-walled parts is shown in Figure 4.

## 3. Path Planning and Machine Tool Motion Simulation of Box-Shaped Thin-Walled Parts

### 3.1. Path Planning of Thin-Walled Parts

The laser deposition path design is crucial in the additive and subtractive manufacturing of box-shaped thin-walled parts. It impacts the surface deformation of the formed part and its processing efficiency. Consequently, minimizing surface deformation while reducing the laser head’s empty walking time is essential to ensure optimal processing efficiency. The deposition path for box-shaped thin-walled parts has multiple right-angle turning points that the laser head must traverse multiple times during the same layer’s deposition process.

This can lead to deformation accumulation at the intersection position, resulting in a higher forming height at the right-angle turning point than other positions when the additive height reaches a certain level. Therefore, it is necessary to avoid redundant deposition of the box-shaped deposition path on the same layer as much as possible. However, not producing repeated deposition will increase the laser deposition head’s empty walking time, which can negatively affect the part’s processing efficiency. Additionally, discontinuous processing requires multiple repeated positioning, which can cause unnecessary processing errors. Therefore, VERICUT 9.0.1 software is used to simulate the deposition path of additive manufacturing to seek a deposition path with high forming accuracy and processing efficiency.

**Figure 5:**
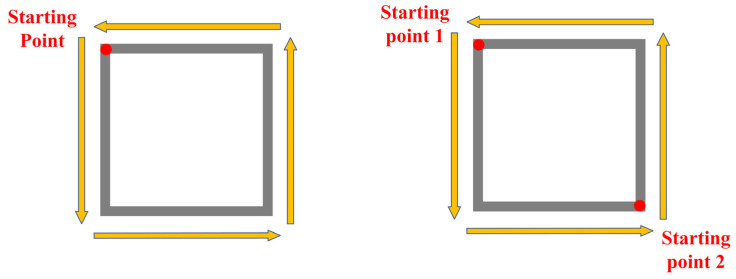
Box-shaped path planning.

Several different processing paths have been designed to address the processing issues of thin-walled box-shaped parts, as illustrated in Figure 5. Path 1 is a simple clockwise deposition, the most straightforward approach. In contrast, Path 2 differs from Path 1 in that the starting points for odd and even layers are diagonal. Starting from the red point 1, the deposition is carried out clockwise. After completing the deposition, the process starts again from point 2 and deposits clockwise around the box, layer by layer. Although this approach accumulates more powder during the deposition process at the starting points, deformation accumulation may also occur in right-angle bends. The upper surface can be milled to eliminate the accumulated deformation during the milling process.

### 3.2. Machine Tool Motion Simulation and Collision Analysis

**Figure 6:**
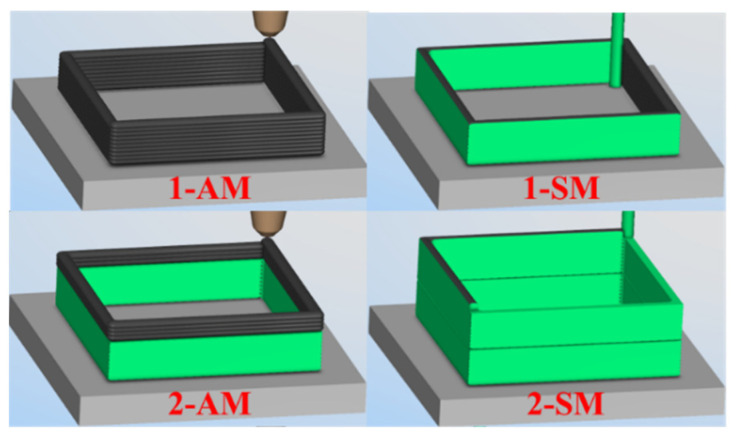
VERICUT simulation of additive and subtractive hybrid manufacturing.

VERICUT simulation was performed on the G-codes of Path 1 and 2, respectively, and the processing efficiency was compared. Figure 6 shows the simulation process of two additive and subtractive hybrid manufacturing cycles for the box-shaped thin-walled part. During the simulation of machine tool motion, the collision check function in VERICUT was turned on. The parts that collided with each other were marked in red. No red area or collision was warning throughout the entire process, indicating that the CNC G-code for the two instances of additive and subtractive manufacturing of the box-shaped thin-walled part was correct.

**Figure 7:**
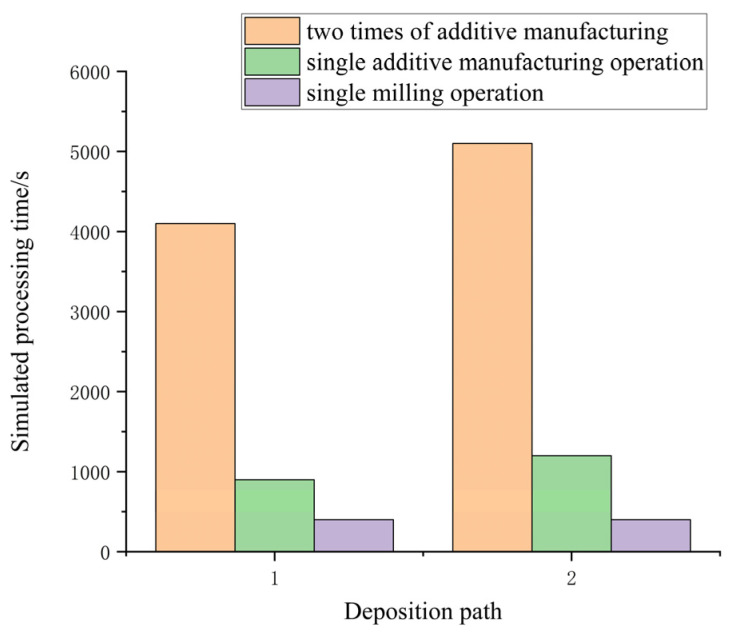
Impact of deposition paths on processing efficiency.

From the simulation results of VERICUT, it is clear that the same shape of the workpiece can be obtained after the subtractive processing regardless of the deposition method of the path, but there is a big difference in the time required for the processing. Since the machining process is slow, the machining time of different paths was counted using the same process and the same resultant animation playback factor (50× speed), as shown in Figure 7. As can be seen from the figure, the machining time of Path 1 is lower than that of Path 2 due to less space-time travel for the deposition of Path 1. In addition, it is obvious to observe that the main reason affecting the processing efficiency in the additive and subtractive hybrid manufacturing process is concentrated in the additive stage. In contrast, the subtractive material has very little effect on the overall processing time. Therefore, additive path optimization should be a more important task in the additive and subtractive material processing process, which is not only related to the processing efficiency of the whole part but also has a certain impact on the deformation of the additive material.

## 4. Experimental Study on Additive and Subtractive Hybrid Manufacturing of 316L Stainless Steel Box-Shaped Thin-Walled Parts

### 4.1. Experimental Materials

The metal powder used in the LMD manufacturing process in this paper is 316L stainless steel (GB standard name: 022Cr17Ni12Mo2). The geometry of the powder particles is a spherical shape, and the main chemical components are C, Si, Mn, Cr, Ni, Mo, and Fe. The particle size ranges from 75 to 150 μm, and the powder has good flowability and thermal processing properties. The chemical composition of the powder is shown in Table 1 [16]. The substrate material used in the melt deposition forming experiment is a 316L stainless steel rolled steel plate with dimensions of 250 × 150 × 15 mm. Before the experiment, the metal powder needs to be dried in a vacuum oven at 100 °C for 4 h to ensure good flowability. Before the additive manufacturing experiment, the surface of the substrate needs to be wiped with industrial anhydrous ethanol to remove oil and dust [28], and the substrate needs to be sandblasted. This is done to reduce laser reflection and energy loss on the one hand and to improve the adhesion of the cladding layer to the substrate on the other hand.

**Figure 8:**
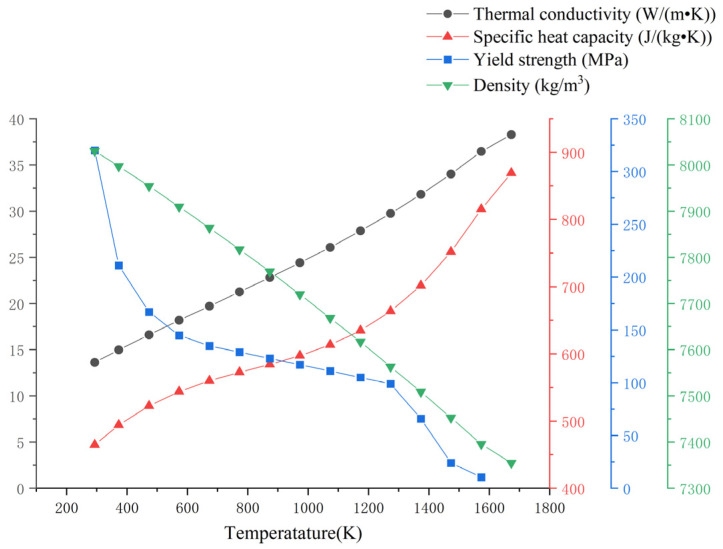
Thermal physical parameters of 316L stainless steel.

The thermal physical parameters of 316L stainless steel at different temperatures are shown in Figure 8 [29]. Despite differences in grain shape between the cladding layer and substrate microstructures during additive manufacturing, the simulation results are minimally impacted by material parameter variations between the additive part and substrate. Therefore, this paper assigns the same material parameters to the cladding layer and substrate, both set as 316L stainless steel, which improves the convergence of finite element calculations. At the same time, the mechanical properties of 316L stainless steel additive parts in actual production are anisotropic [30,31]. However, obtaining basic parameter data for anisotropic 316L materials is difficult. Therefore, the thermodynamic simulation of this model is simplified as isotropic [32].

### 4.2. Experimental Equipments

The additive and subtractive hybrid equipment is a modified additive and subtractive processing machine formed by adding an additive manufacturing system and related auxiliary systems to an existing machine tool.

In the experimental system, the laser emitter is a YLS-2000 fiber laser produced by IPG, with a maximum output power of 2000 W. The laser has advantages, such as high beam quality and high energy utilization, making it suitable for laser-cladding-forming experiments. The powder feeder is an RC-PG-FD dual-barrel powder feeder produced by Nanjing Zhongke Yuchen, which delivers a stable powder flow, precise powder delivery, low noise and vibration, and a size range of 45–149 µm for conveying metal powder. The powder delivery rate can be controlled by setting the rotation speed of the powder delivery disk during processing operations, and metal powder can be continuously and stably delivered to the laser cladding head under reasonable carrier gas flow rate parameters. The YC52 coaxial powder feeding laser cladding head produced by German company PRECITEC has a compact and sturdy structure suitable for fiber lasers with high output power. The laser cladding head is equipped with a ring-shaped nozzle, which can accurately converge the metal powder flow within the focal point of the laser beam to achieve negative defocusing and improve the quality and efficiency of laser additive manufacturing.

In the additive and subtractive hybrid manufacturing detection process, a high-precision HG-C1030 laser displacement sensor produced by Panasonic Corporation of Japan was used to detect the surface deformation of the formed parts during the hybrid manufacturing process. The measurement range is ±5 mm, with a measurement center distance of 30 mm and an accuracy of 10 μm.

### 4.3. Experimental Plan

An additive and subtractive manufacturing process experiment was conducted on the box-shaped thin-walled part with the process parameters shown in Table 2. The deposition tests were carried out using Path 1 and Path 2, and a milling operation was performed after 20 layers of deposition. A laser displacement sensor was used to collect the deformation data of the workpiece at different stages. Deformation refers to the deviation from the nominal outer surface, with the reference surface defined as the distance from the center of the melt pool to the laser displacement sensor. Specifically, the reference surface distance is calculated by subtracting half of the melt pool width from the measured distance between the melt pool center and the laser displacement sensor. In the deposition of the box-shaped thin-walled part, in addition to avoiding the problem of multiple pauses, attention should also be paid to the machining of right-angle bends. The machine tool undergoes acceleration and deceleration when performing right-angle deposition, leading to overheating at the right angle, resulting in excessive stress or even cracking of the part. To address this issue, the arc interpolation command of the machine tool is used to replace the box right-angle bend with a small radius arc path. This circular arc transition allows the laser head to process continuously at the corner without accelerating and decelerating, effectively avoiding excessive deposition at the corner.

### 4.4. Experimental Results

**Figure 9:**
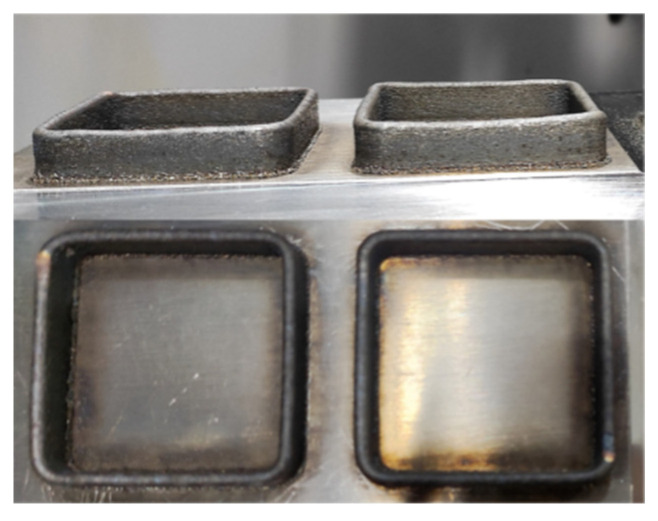
Additive box-shaped thin-walled parts under two different paths.

To address the box deposition problem, a box additive manufacturing experiment was conducted with a side length of 50 mm and 20 layers deposited. Figure 9 displays the box additive formed parts of Paths 1 and 2 (from left to right), respectively. From the top view, the box shapes of both paths are relatively regular, and no obvious defects appear. The deposition of Path 1 is continuous, and the box thin-walled part does not repeat the trajectory at the end of the previous layer when depositing the next layer but continues to deposit along counterclockwise. Therefore, the whole process does not show obvious thick ends or a thin middle. Path 2 has two starting points, and the odd and even layers start from the box diagonal. The forming parts show that the deposition height at the starting point of the two corners is higher than the other two corners, indicating that more powder will be deposited at the starting position, resulting in a thicker workpiece. Therefore, Path 1 should be selected for processing for additive manufacturing of box-shaped thin-walled parts.

**Figure 10:**
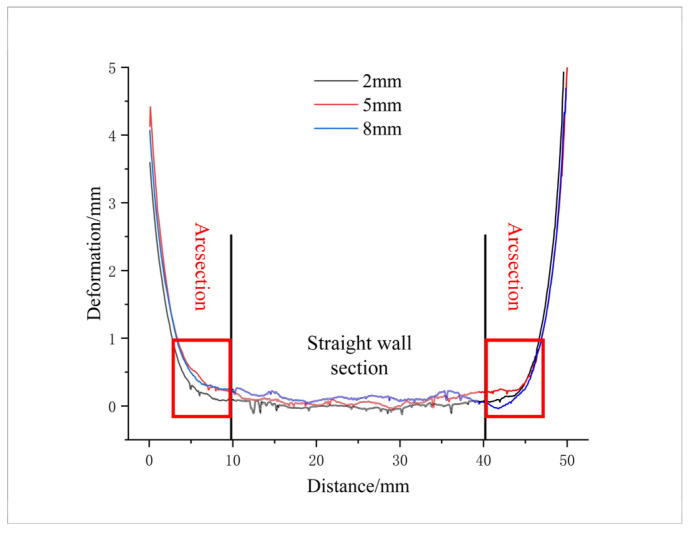
Box-side deformation data.

**Figure 11:**
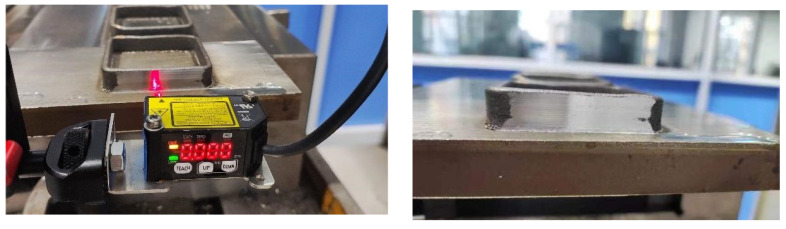
Milling of box-shaped, additive-manufactured thin-walled parts.

**Figure 12:**
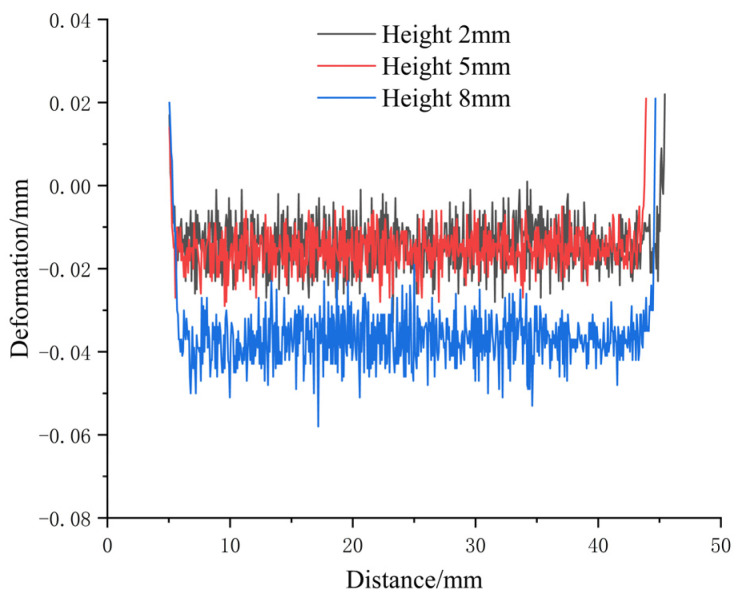
Surface deformation data after milling of additive parts.

A laser displacement sensor was used to measure the surface deformation of the box-shaped thin-walled part produced using Path 1. The lateral deformation data at heights of 2 mm, 5 mm, and 8 mm on one side of the part were extracted, as shown in Figure 10. The deformation can be divided into the straight wall and the arc. Overall, the deformation increased slightly with height, and a sharp increase in the curve was observed outside of the arc, indicating that some deformation occurred in the adjacent two sides. The maximum deformation of the straight wall was 0.198 mm, and the maximum deformation of the arc was 0.241 mm. After the additive measurement was completed, the part was directly milled. The surface deformation data were measured again, as shown in Figure 11, the surface of the box-shaped additive after milling appears to have a noticeable metallic luster. Figure 12 shows the surface deformation data after milling, the deformation is more obvious at the height of 8 mm than at the height of 5 mm and 2 mm, and the maximum deformation is 0.057 mm. This may be due to the greater vibration of the thin-walled part near the top during milling. Compared to the surface after additive manufacturing, the maximum deformation was reduced by 76.3%, indicating that milling effectively improved the surface quality of the part.

## 5. Finite Element Simulation of 316L Stainless Steel Box-Shaped Thin-Walled Parts Manufactured by LMD

### 5.1. Finite Element Model Establishment

#### 5.1.1. Theoretical Research on Simulation of LMD Process

The indirect coupling field analysis method is utilized to conduct nonlinear transient thermal analysis, followed by stress analysis based on the thermal analysis results. Sequential coupling can be alternately performed between different physical fields until convergence to a desired level of accuracy is achieved. Solid70 and Solid185 elements are used in the study. Solid70 is a low-order element consisting of eight nodes with heat transfer capability in all three directions and is utilized for thermal analysis. Solid185 is a relatively high-order solid element, with eight nodes in each element with three degrees of freedom each. It possesses the ability of hyperelasticity, stress hardening, and large deformation and can be used as a stress analysis element after temperature analysis.

As additive manufacturing involves a process of continuous deposition, the birth–death element technique commonly used in simulations currently has discontinuities. To achieve better simulation results for this process, it is necessary to use a small enough mesh size and time step. However, a small element size and a large number of time steps can result in a huge computational cost. Therefore, the mesh size of this study’s additive manufacturing simulation elements is set to be less than 1 mm. Additionally, an interpolation method is utilized to read the result files to ensure the effectiveness of the simulation without exceeding the expected computational scale.

ANSYS provides a command to read the temperature field as a load, dividing the temperature field simulation into multiple segments. Only the area near the processing zone is refined for meshing during each segment simulation, while the mesh size in other areas can be adjusted appropriately. The temperature distribution at the end of the last simulation is read and used as the initial temperature field for the next simulation, ensuring the continuity of the temperature simulation. In additive manufacturing simulations, the 2D Gaussian heat source is a heat source model that closely approximates the actual laser energy distribution. In this study, the maximum heat flux density at the center of the laser spot is given by Equation (2).

Therefore, the maximum volumetric heat generation rate is given by:

(17) $$HGENm=\frac{q_{m}}{L}$$

Combined with the Gaussian function, the volumetric heat generation rate is distributed in a Gaussian shape within the element, so the Gaussian heat source used in the numerical simulation is defined as Equation (3) above.

A universal fixture on a CNC machine is used to fix the substrate during the deposition process to prevent the workpiece’s high-temperature oxidation from affecting the performance of the formed parts. The laser head has a protective gas to spray onto the deposition area. For ANSYS numerical simulation, different heat dissipation boundary conditions are set for each part. To simplify the model, the fixture is not included. The area near the laser head experiences high temperatures due to the forced convection of gas and heat radiation exchange. Therefore, the heat radiation coefficient is relatively large in this region. Additionally, the gas convection intensity is highest near the laser melting head. Parts farther away from the laser head experience lower temperatures as gas convection intensity decreases with increasing distance, resulting in lower heat radiation intensity. Finally, the substrate’s bottom and two ends are furthest from the laser head, resulting in natural convection dominating these areas with a convective coefficient typically around 20.

For the part, the internal thermal conductivity coefficient is determined by the material properties, while the heat radiation and forced convection are based on the convective function related to the number of additive layers, as shown in Equation (18):

(18) conv=RE+RT×(N−1)

where RE is the initial heat dissipation coefficient, expressed in the text as the natural or forced convection coefficient; RT is the varying heat dissipation coefficient, which in this paper is the modified value of the thermal radiation coefficient and forced convection coefficient; and N is the number of deposited layers of the workpiece.

#### 5.1.2. Finite Element Model

**Figure 13:**
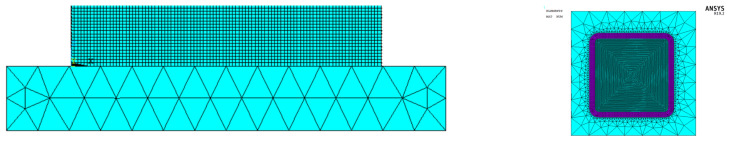
Finite element model of the box-shaped thin-walled part.

Due to the simplicity of the geometry model, the geometric modeling of the box-shaped thin-walled part and the deposition substrate was directly carried out in the ANSYS 19.2 classic interface Mechanical APDL, as shown in Figure 13. The substrate size is 80 mm × 80 mm × 10 mm. In the simulation of additive and subtractive manufacturing, the main focus is on the deposition part, and the large geometric size of the substrate will bring unnecessary and large-scale computational costs. Therefore, the mesh size of the substrate is set to 5 mm, and the mesh size of the connection between the substrate and the workpiece is refined. The mesh of the deposition part should be dense and not larger than 1 mm; given this, after multiple comparisons, a mesh size of 0.5 mm was chosen. Before the next additive manufacturing, the part will be remodeled to simulate the state of the workpiece after material removal during milling, and the heat dissipation conditions need to be reassigned to the new model to ensure consistency between the model and the experiment.

**Figure 14:**
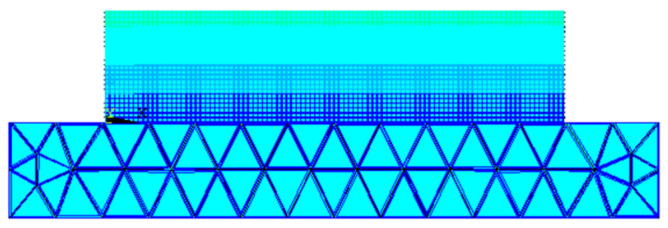
Set heat dissipation conditions for the box.

The closer the box is to the laser head, the greater the heat dissipation coefficient, which is mainly from thermal radiation and convection. On the other hand, the heat dissipation of the part farther away from the laser head and closer to the substrate is mainly through thermal conduction and convection. Therefore, the heat dissipation coefficient in different areas is also different. It should be noted that because the surface inside the box is in a relatively closed space, the heat dissipation effect of the outer surface of the box is better than that of the inner surface during the deposition process of the box. Figure 14 shows the heat dissipation settings of the outer surface of the box and the substrate. The heat dissipation from the substrate to the top of the box gradually changes from blue to yellow–green, representing a greater heat dissipation coefficient the higher up the box is, and the heat dissipation coefficient is linearly related to the deposition height.

### 5.2. Additive Manufacturing Simulation of Box-Shaped Thin-Walled Parts

#### 5.2.1. Temperature Field Analysis

Numerical simulations of additive manufacturing with different deposition paths are conducted to find the temperature and deformation distribution law, select the optimal path, and predict the stress and deformation.

(1) Path 1

**Figure 15:**
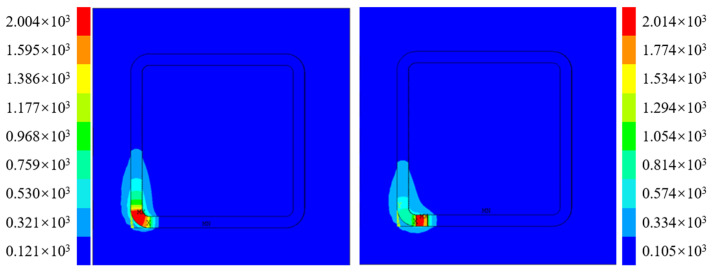
Nephogram of additive temperature for Path 1.

As shown in Figure 15, the process of the arc transition zone and interlayer transition for Path 1 is displayed. The blue represents the substrate and the processed parts, and the units of the unprocessed parts have not been activated and have no practical significance.

When the laser scans the arc area at a constant speed, it can be seen that the temperature is 2004 °C. When the next layer is deposited, the laser continuously scans at the other end adjacent to the arc; the temperature of the molten pool is 2014 °C. Although the temperature has increased slightly compared to the arc area, the difference is relatively small. This is because the laser continuously scans and there is no deceleration, so the temperature is relatively stable.

(2) Path 2

**Figure 16:**
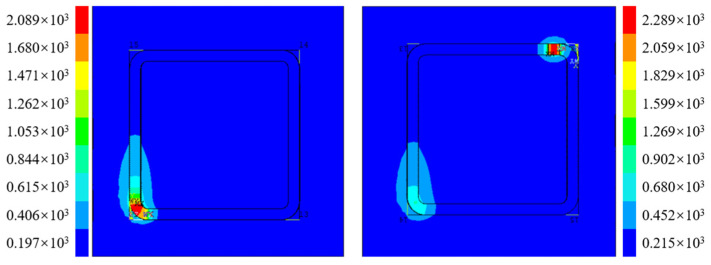
Nephogram of additive temperature in Path 2.

As illustrated in Figure 16, the temperature field of the arc region processing and interlayer region processing of Path 2 shows that the previous layer processing ends with the arc region, and the highest temperature at this point is 2089 °C. However, in practice, there is a time interval between the completion of the last-layer processing and the start of the next-layer processing, during which the laser head moves to the diagonal position. To simulate the temperature of the workpiece during laser head movement, a load step with no heat source is applied in the simulation and set to natural cooling. At the start of the next layer, a certain amount of time is required for the machine tool to start up and stabilize. As a result, the laser’s moving speed is reduced accordingly in the load step at each laser starting point position. Consequently, the highest temperature at the starting point is 2289 °C, which is significantly higher than the molten pool temperature at other times.

**Figure 17:**
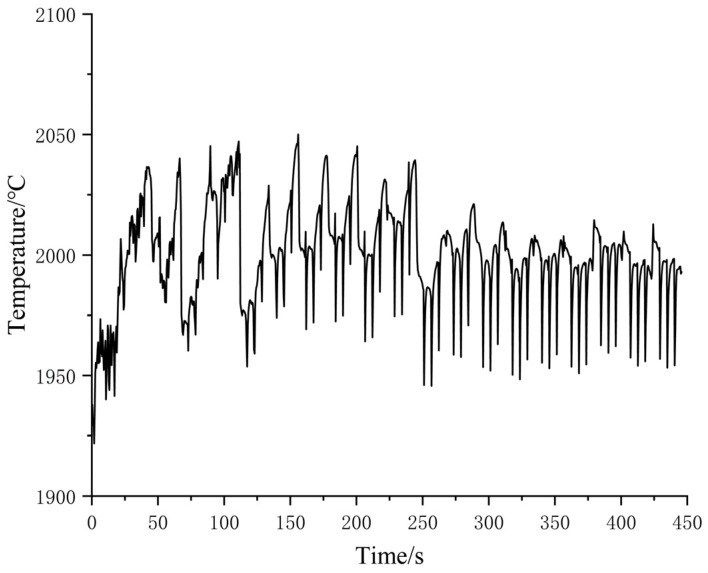
Path 1 melt pool temperature.

Based on the above analysis, it is apparent that for Path 1, the heat deposition effect caused by the laser start–stop is almost non-existent due to continuous processing. Therefore, further simulation of 20 deposition layers was carried out for Path 1. To better illustrate the temperature changes during the entire processing process, the highest temperature at each moment was extracted from the simulation to draw a temperature–time curve, as shown in Figure 17. The curve clearly shows that the temperature undergoes small fluctuations at regular intervals during the processing process. Each layer processes four arc transition areas, which tend to have a certain temperature increase trend. During arc motion, the laser spot moves slower on the inner diameter than the outer diameter, resulting in temperature fluctuations. These fluctuations are reflected in the temperature–time curve, which shows a periodic pattern of small fluctuations.

In addition, the curve illustrates that there is a certain temperature rise during interlayer processing. This is due to the absence of cooling time after the previous layer, resulting in heat accumulation and temperature rise during the next layer processing. However, this temperature accumulation is much smaller than the temperature accumulation at the starting point of other paths. These phenomena are consistent with the temperature field described above. Overall, the additive molten pool temperature of the box-shaped thin-walled part is relatively regular, and the highest temperature at most moments is stable at around 2000 °C. The periodic temperature changes can also predict the distribution of stress and deformation to a certain extent.

#### 5.2.2. Stress and Deformation

**Figure 18:**
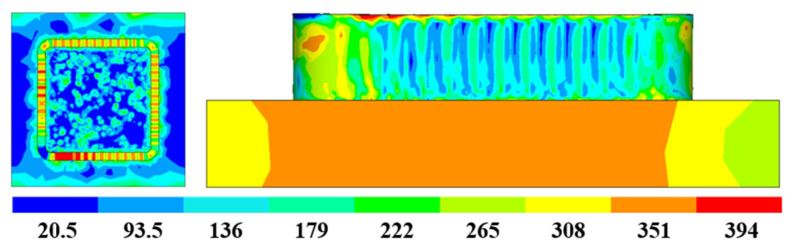
Additive residual stress distribution of path 1.

**Figure 19:**
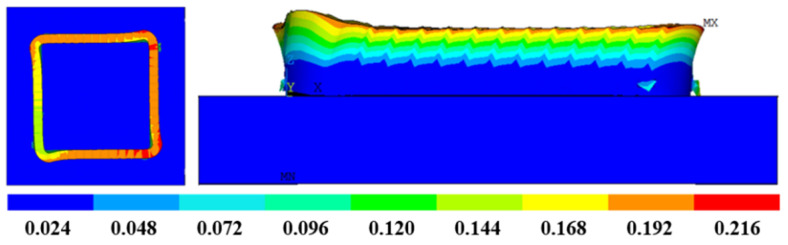
Additive deformation nephogram of path 1.

Figure 18 shows the thermal stress distribution results of Path 1 in a box-shaped structure. The stress distribution is relatively uniform, but many stress concentration areas also exist. The lateral stress distribution shows that the area closer to the arc of the box-shaped thin-walled part exhibits higher stress, while the middle area has lower and more stable stress. From a top-down view, the top of the thin-walled part is the area with the highest stress, and the maximum residual stress is approximately 394 MPa. Figure 19 shows the additive deformation simulation of the thin-walled part in Path 1. To display the deformation situation intuitively, the magnification factor of this deformation nephogram is set to 10. The box-shaped thin-walled part in the figure shows obvious deformation and distortion with greater bending outward at higher deposition heights. The larger deformation area is mainly in the arc area, and the maximum deformation amount is 0.216 mm. The deformation trend was similar to the experimental results, with a difference of 0.025 mm in the maximum predicted value.

**Figure 20:**
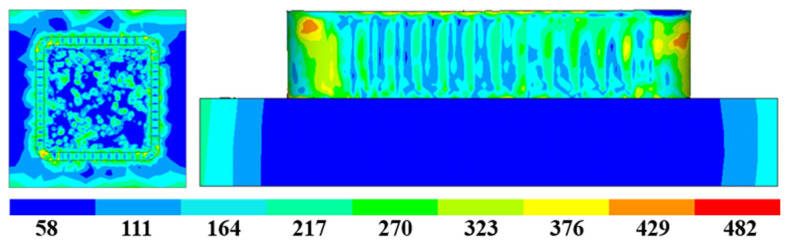
Additive residual stress distribution of Path 2.

**Figure 21:**
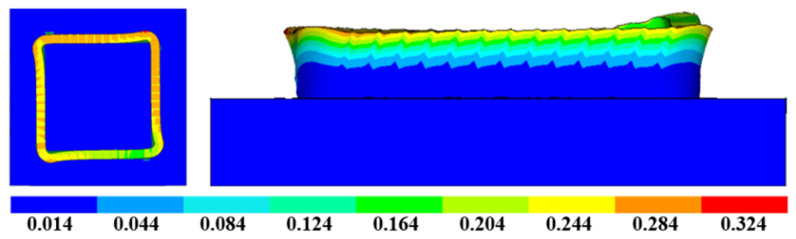
Additive deformation nephogram of Path 2.

The stress distribution of Path 2 is shown in Figure 20. Since there are multiple changes of starting points for Path 2, the temperature fluctuation is relatively large, resulting in a larger residual thermal stress of the additive component in Path 2. The maximum residual stress is 482 MPa, and the residual stress distribution has a certain regularity. The higher the stress, the closer to the top the arc and the two additive starting points are. The stress distribution in other parts is relatively uniform, averaging around 200 MPa to 300 MPa. The deformation of the box-shaped thin-walled part is related to the distribution regularity of residual stress. Figure 21 displays the deformation distribution of the additive component in Path 2. It is obvious that the tendency of the box to twist outward gradually increases with the increase in height. The deformation at the arc is relatively obvious, but the deformation in the arc area where the two starting points are located is larger than the other two arc areas at the same height. The maximum deformation amount is 0.324 mm, which is significantly larger than the maximum deformation amount in Path 1. In addition, if excessive powder feeding due to low speed at the starting point position is considered, the deformation will be further increased.

## 6. Deformation Prediction of Additive and Subtractive Hybrid Manufacturing for 316L Stainless Steel Box-Shaped Thin-Walled Parts

**Figure 22:**
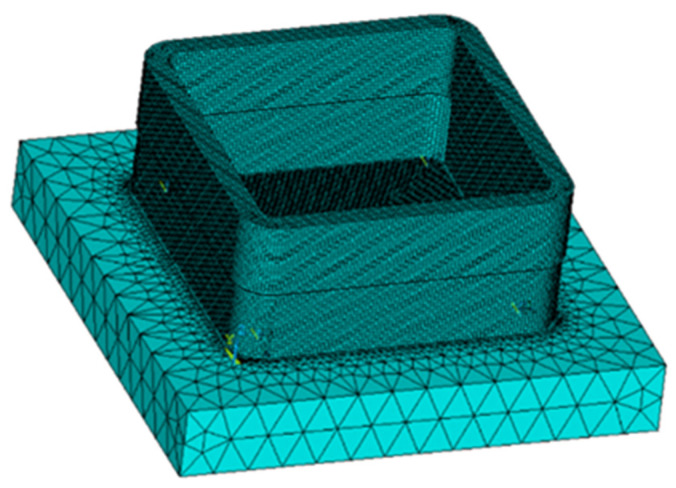
Box finite element model.

In the above study, path planning of the box-shaped thin-walled part, machine tool motion simulation, and additive manufacturing simulation of the box-shaped thin-walled part were conducted. It was found that the first path planning had the best effect on the box-shaped additive forming, and the finite element model of the box-shaped part was validated through a first-stage additive and subtractive hybrid manufacturing experiment, demonstrating the effectiveness of the box-shaped finite element model. Therefore, based on the above box-shaped simulation, a simulation prediction of the two-stage additive and subtractive hybrid manufacturing of the box-shaped thin-walled part was carried out. Figure 22 shows the finite element model of the two-stage additive manufacturing of the box-shaped thin-walled part. In the second simulation, the elements milled in the previous stage were “killed” before calculation.

**Figure 23:**
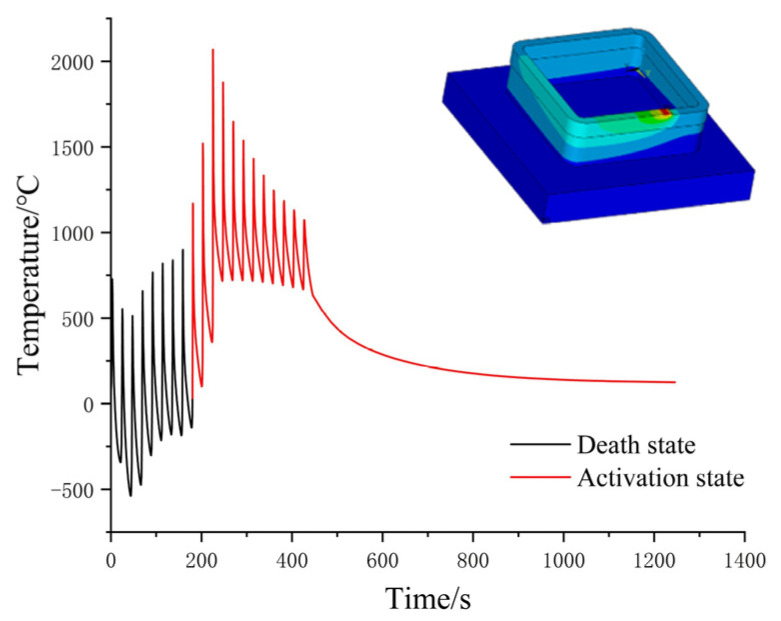
Temperature–time change history of nodes.

Figure 23 shows the temperature profile of a node during the second stage of additive manufacturing. The black part of the curve represents the “dead” state of the node, while the red part indicates that the node has started to activate and enter the processing stage. The node temperature decreases periodically, and the peak value gets lower until the entire part undergoes natural cooling until it reaches room temperature. This periodic change is also the main source of residual stress during the additive process. The highest temperature experienced by the node was around 2000 °C, which exceeded the melting point of 316L stainless steel.

**Figure 24:**
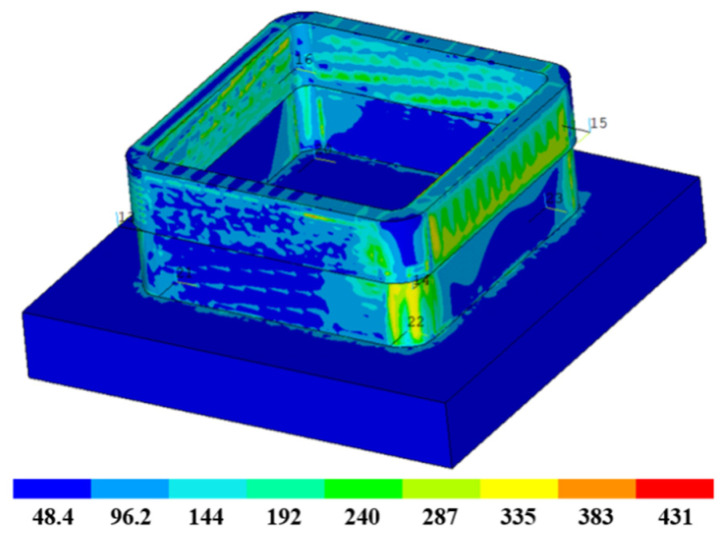
Residual stress and deformation nephogram of box-shaped thin-walled part.

The residual stress and deformation distribution (enlarged by 10 times) of the second box-shaped thin-walled part is presented in Figure 24. The overall stress distribution of the box-shaped part is relatively uniform after the second additive manufacturing, with slightly higher stress in the transition area between the first and second stages. This is attributed to the flow effect at both ends, which is caused by the width of the second additive melt path (3 mm) exceeding the thickness after the first milling (2 mm).

The maximum residual stress after the second stage of additive manufacturing is 431 MPa, which is slightly higher than the first stage. In addition, from the stress distribution, the second stage of additive manufacturing heated the first stage part and changed its stress state, reducing the maximum residual stress of the first stage. Regarding deformation, the box-shaped thin-walled part collapses downward in the arc additive manufacturing area in the second stage, with some concave deformations on the four sides. The deformation is more pronounced near the arc, with a maximum deformation of 0.63 mm.

**Figure 25:**
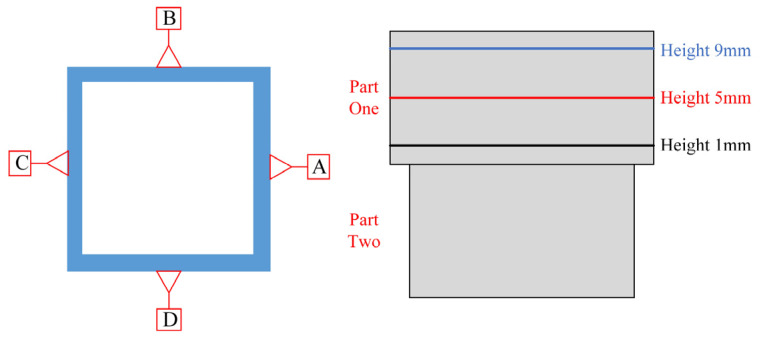
Schematic diagram of the second segment deformation data extraction.

**Figure 26:**
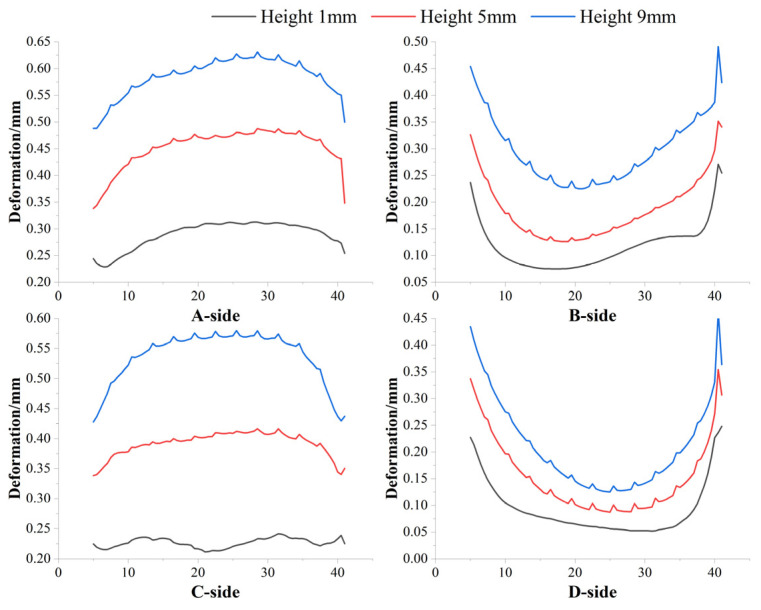
Deformation data of the four sides in the second section of additive manufacturing.

To provide a more intuitive representation of the deformation of the four sides of the box-shaped thin-walled part, a schematic diagram of data extraction for the box-shaped thin-walled part is presented in Figure 25. Deformation data at heights of 1 mm, 5 mm, and 9 mm were extracted from the A, B, C, and D sides, respectively, in the second section, as shown in Figure 26.

Based on the deformation data of the four sides, it can be seen that the four sides of the box-shaped thin-walled part show a trend of increasing deformation with the increase of deposition height. This indicates that the second section of the additive part also exhibits a similar pattern to the first section, where the higher the deposition height, the more the box-shaped part bends outward. This may be due to the fact that the higher the deposition height, the greater the cumulative temperature and the corresponding increase in thermal stress. This occurs in the inner surface, wherein the higher the deposition height, the more the box-shaped part bends outward. Thin-walled parts are in a relatively closed space, so the higher the deposition height of the outer surface, the more outward bending of the box shape; it has better heat dissipation than the inner surface. This causes the outer surface to contract faster, causing the thin-walled part to twist outward as the deposition height increases.

**Figure 27:**
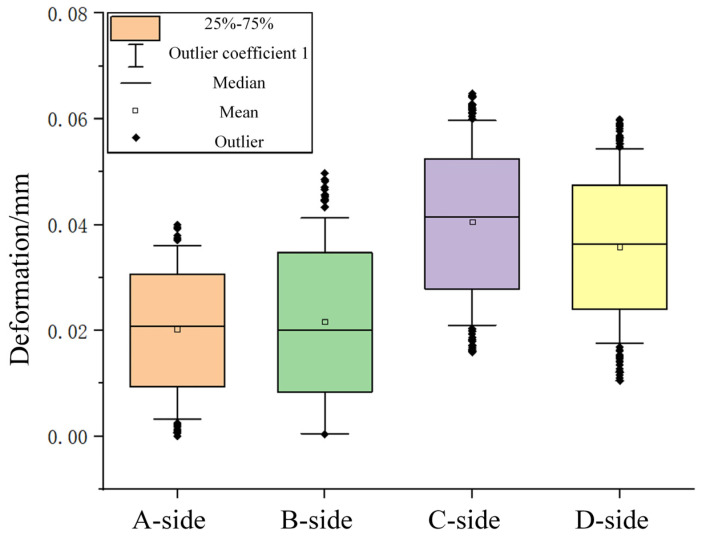
Surface deformation after milling.

In addition, the surface deformation data after milling was extracted from four faces at a height of 5 mm. The box line diagram plotted in Figure 27 shows that the maximum deformation was less than 0.06 mm, which was reduced by more than 90%, indicating that milling improved the surface quality after the additive process.

## 7. Conclusions

This paper used the additive and subtractive hybrid manufacturing machine tool to carry out the additive and subtractive hybrid manufacturing process experiment of the box-shaped thin-walled parts of 316L stainless steel, collected the relevant experimental data, and used ANSYS 19.2 software to establish the thermal–mechanical finite element model of additive and subtractive manufacturing; the temperature change of the additive process and the deformation of the formed parts were numerically simulated, finishing the summary of the experiment and simulation results of this paper. The following conclusions were obtained:

(1) According to the LMD process simulation theory, a simulation model for additive manufacturing of box-shaped thin-walled structures was established, predicting the temperature field, stress, and deformation of box-shaped thin-walled structures after laser additive manufacturing. The experimental results were generally consistent with the simulation prediction trend, with a difference of 0.025 mm in the predicted maximum deformation.

(2) Using this model, simulation predictions were conducted for a box-shaped thin-walled structure with two segments of additive and subtractive manufacturing. For the temperature field, a point on the outer surface of the thin-walled part was selected, and the time–temperature profile of the point was obtained. The temperature increase process had a gradually increasing peak before the arrival of the laser heat source. After the arrival of the heat source, the temperature peak decreases, and the temperature fluctuation gradually stabilizes. The temperature drop curve approximates one of the inverse proportional function curves.

(3) For stress and deformation, the second segment’s processing caused the first segment’s workpiece to be heated and change its stress state, reducing the maximum residual stress of the first segment. The closer the second segment’s thin-walled structure is to the arc, the greater the deformation, with a maximum deformation of 0.63 mm. At a height of 5 mm, the surface deformation was extracted after milling on all four sides, with a maximum deformation of less than 0.06 mm, which was reduced by more than 90%, indicating that milling improved the surface quality after additive manufacturing.

In this paper, a series of experiments were carried out for the alternating hybrid manufacturing of additive and subtractive materials to study the additive and subtractive hybrid manufacturing process of the thin-walled parts of the box, as well as the deformation of the various stages of the law, to obtain some of the important defects affecting the distribution of the residual stress, deformation of the size of the important defects, and to predict the deformation of the thin-walled parts of the box; however, there is still a part of the problem that needs to be solved.

Although a finite element model was established for numerical simulation in the study, there are still certain limitations. The model only considers the starting position and low scanning speed during the deposition process without accounting for excessive powder feeding, leading to inadequate control over deformation. Additionally, thermal conditions play a significant role in the simulation, affecting temperature distribution, stress field results, and deformation. While a linear thermal condition was established in this paper, in reality, the thermal conditions at different nodes vary with time. Therefore, a more accurate thermal model can be developed in future research to improve the simulation accuracy.

## Figures and Tables

**Table 1:** Chemical composition of 316L stainless steel powder used in AM experiments.

Chemical Composition	C	Si	Mn	P	S	O	Ni	Cr	Mo
(wt%)	0.025	0.53	1.46	0.01	0.015	0.53	11.78	16.69	2.41

**Table 2:** Laser additive manufacturing process parameters.

Equipment	Adjustable Parameters	Experimental Parameter Values
laser	laser power (W)	1200
powder feeder	powder delivery rate (g/min)	6.271
protective gas system	carrier gas pressure (MPa)	3.5
	protective gas delivery pressure (MPa)	3.5
laser cladding head	spot diameter (mm)	2
machine tool	Z-axis lift (mm)	0.5
	inter-layer scan interval time (s)	continuous scanning
	feed rate (mm/min)	400

## Data Availability

The data that support the findings of this study are available from the corresponding author upon reasonable request. The data are not publicly available due to privacy or ethical restricitions.
